# Suppliers’ Perspectives on Cage-Free Eggs in China

**DOI:** 10.3390/ani14111625

**Published:** 2024-05-30

**Authors:** Maria Chen, Huipin Lee, Yuchen Liu, Daniel M. Weary

**Affiliations:** 1 Animal Welfare Program, Faculty of Land and Food Systems, University of British Columbia, 2357 Main Mall, Vancouver, BC V6T 1Z4, Canada; maria.chen1994@gmail.com (M.C.); hplee6@student.ubc.ca (H.L.); 2 Institute of Finance and Economics, Shanghai University of Finance and Economics, Shanghai 200433, China

**Keywords:** egg producers, egg suppliers, qualitative research, animal welfare, consumers

## Abstract

**Simple Summary:**

Promoting cage-free eggs supports a housing system which may benefit the lives of egg-laying hens. China is the world’s largest producer and consumer of eggs, and most of these eggs are produced in caged systems. Our aim was to understand the promotion strategies of companies supplying cage-free eggs in China, through examining 10 companies selling these products to business and consumer buyers. We found that the companies mainly promoted their eggs through considerations of price (for example, by engaging buyers who would pay more), attributes of the egg which buyers can sense (for example, accommodating buyers’ preferences for better taste and specific shell colors), and improving buyers’ understanding of and trust in non-sensory credence attributes (for example, through communicating to buyers about cage-free production and using trusted certifications). Our study describes strategies of cage-free egg promotion that may also help inform promotion of other products with the potential to improve animal welfare.

**Abstract:**

Successful promotion of cage-free eggs supports a housing system offering potential for improved hen welfare. As the world’s largest egg producer and consumer, China offers much potential for welfare improvements. We examined 10 Chinese companies supplying cage-free eggs (four using indoor systems, six with outdoor access) to understand their strategies to promote cage-free eggs to businesses and consumers. We purposively sampled 12 employees from these companies familiar with production or sales. We conducted two–three semi-structured interviews per participant, collected public online documents (including online shops and social media content), and recorded field notes. We analyzed the data using template analysis to generate key results. Participants reported buyers being unfamiliar with ‘animal welfare’ and ‘cage-free’, but familiar with concepts associated with ‘free-range’. Participants considered three attributes when promoting cage-free eggs: price (engaging buyers who were willing to pay more), experiential attributes (e.g., taste, accommodating buyer preferences), and non-sensory credence attributes (e.g., cage-free production, improving buyers’ understanding and trust). Our results are not generalizable, though they may be transferable to similar contexts. Understanding how companies promoted cage-free eggs to buyers may help inform promotion of other animal products with welfare attributes. Simultaneous efforts are needed to ensure actual welfare improvements on farms.

## 1. Introduction

Domestication and husbandry of chickens in China dates back to 3000 BCE [1], but before the 1970s, chickens often lived in small-scale free-range systems and were often indigenous breeds [2]. Increasing flock sizes and the use of cages became common in the 1970s after the introduction of Reform and Opening Up Policies [2]. Since 1985, China has been the world’s largest producer and consumer of eggs, with 3.2 billion hens producing 40% of the world’s eggs in 2021 [3]. The majority of the hens producing these eggs live in cages; the International Egg Commission estimated that approximately 90% of eggs are produced using caged systems, 9% are free-range, and 1% are from cage-free indoor systems [4].

Public concerns in Europe surrounding farm animal welfare increased in the 1960s, in response to the rise in restrictive housing systems for farmed animals like layer hens [5]. Such concerns were reflected in animal advocacy and scientific inquiry into improving layer hen housing, leading to changes in policy in more than 30 countries (e.g., the EU Directive 1999/74/EU banned conventional cages in member countries by 2012) and changes in market demand and supply (e.g., global companies committing to sourcing only cage-free eggs; [6]).

In China, there have been increasing discussions about ‘cage-free’ (非笼养) production among stakeholders, including NGOs, consultancies, academia, equipment manufacturers, suppliers, processors, and retailers [7,8], with resulting development of cage-free industry standards and labelling schemes [9]. ‘Cage-free egg’ is an English term translated into Mandarin as ‘非笼养鸡蛋’ (literally ‘non-cage raised chicken egg’). In the first industry paper about cage-free eggs, ‘cage-free egg’ was defined as eggs laid by hens living in environments without cages, including indoor ‘barns’ (舍内平养), ‘free-range’ (自由散养), and ‘pasture free-range’ (山林/牧场散养) systems that allow hens to move freely, forage, access resources such as litter, perches, and comfortable nests [8]. Within the egg industry, the term ‘cage-free’ is often associated with the concept of better animal welfare; the term ‘welfare egg’ (福利鸡蛋) is used synonymously with ‘cage-free egg’, for example, in industry reports [8], working groups [7], and conferences (e.g., at the five Quality and Welfare Egg Conferences; [10]). 

Market demand from consumer and business buyers can shape production methods, especially in the absence of regulations mandating cage-free production [6]. There are no official market data on cage-free products, and the lack of egg packaging and fraudulent marketing claims make it difficult for consumers to identify these products. In 2021, Consultancy Lever estimated that more than 17% of packaged egg products in 10 supermarkets from first-tier Chinese cities were marketed with terms (e.g., free-range ‘散养’, cage-free ‘非笼养’, organic ‘有机’) or pictures suggesting cage-free environments [8]. Previous research in China has described consumer preferences for free-range eggs [11,12]. Although industry conceptions of cage-free include free-range production, consumers do not always understand this relationship; in a survey conducted by Consultancy IQC in 2020, 40.5% of participants in China reported purchasing ‘free-range’ eggs, while only 6.3% reported purchasing ‘cage-free’ eggs. Furthermore, 80% of participants reported being unsure what ‘cage-free’ eggs are, and one-third reported that they had never previously heard the term [8]. 

Cage-free eggs are one example of a product in the Chinese market explicitly associated with animal welfare, and thus offer an opportunity for engaging consumers with the concept of animal welfare [13]. There is increasing public awareness of the term ‘animal welfare’ [14,15]. Sinclair et al. [16] found that 72% of Chinese consumers surveyed agreed that egg-producing chickens should not suffer, and 65.5% preferred to buy eggs from cage-free hens. Beyond individual consumers, more than 50 companies with international headquarters have committed to sourcing cage-free eggs in China [8], and Chinese-owned companies such as Zoo Coffee, City Shop, and City’super also made this commitment [17,18]. Consultancy Lever estimated around 1.2 million cage-free eggs are needed each year to fulfill these corporate commitments [19].

Despite some demand for cage-free (including free-range) eggs, suppliers within the supply chain express challenges associated with cage-free production. Yang [20] interviewed Chinese producers using caged systems and found that they generally held negative views towards cage-free production, citing concerns regarding hen health, productivity, profitability, and food safety; these producers also associated cage-free systems with challenges, including lack of consumer willingness to pay, lack of land, strict environmental protection policies, and difficulty in managing cage-free production. De Luna et al. [21] surveyed 22 Chinese producers of caged systems and found they chose caged systems due to reduced cost, land optimization, ease of management, scalability, and lower labor cost; only 14% of these participants viewed cage-free production as feasible. However, these producers were able to identify some reasons for adopting cage-free production, including higher animal welfare, increased buyer demand, improved product quality, and access to higher prices. 

Examples of successful cage-free production exist in many regions of the world [22], and He et al. [23] found with proper management, some free-range farms in China can be more profitable than caged farms. Beyond ensuring production, the successful marketing of egg products is crucial for supporting this production. Egg suppliers interact with a variety of stakeholders, including consumers and business buyers, and make decisions that directly affect the lives of hens in their care. The aim of our study was to describe perspectives of suppliers in China who produce cage-free eggs to understand what strategies they used to promote these eggs.

## 2. Materials and Methods

### 2.1. Multi-Case Study

To understand strategies for marketing cage-free eggs in China, we conducted a multi-case study of 10 companies selling into this market. According to Merriam, the defining characteristic of case study research is “the object of study: the case”, which is “a single entity, a unit around which there are boundaries” [24]. Case studies “focuses on holistic description and explanation” [24] of a phenomenon, in this case, the marketing of cage-free eggs in China. Multi-case studies involve the use of multiple cases and allow comparisons between cases. 

### 2.2. Case and Participant Sampling

As Yang described, China’s egg industry can be characterized by its diversity, including in layer genetics, egg products, farm scale, and housing systems [2]. To capture this diversity, we used maximum variation sampling [24,25], where we purposefully selected diverse companies selling eggs produced in cage-free systems in China between 3 July and 1 November 2022. Maximum variation sampling helps “document variations that have emerged in adapting to different conditions” [26]. The inclusion criterion for companies was that the company must be currently producing and selling eggs produced from cage-free systems in China (including any system where hens are kept outside of cages, e.g., indoor barns with or without outdoor access). The dimensions in which we sought diversity among companies included hen housing (4 indoor, 6 outdoor systems), total flock size (6000 to 110,000 hens), hen breeds (local and foreign breeds), distribution channels (from companies focused on direct consumer sales to companies prioritizing selling to business clients), and marketing efforts (from minimal product packaging and marketing to managing a marketing and sales team). Participant background also varied, ranging from production to marketing; participant experience in industry ranged from 2 to 40 years. Data were anonymized to protect participant identity, with companies anonymized as Companies A–J, and individual participants identified using a code (e.g., participant A1 refers to Participant 1 from Company A). 

We recruited 8 diverse companies from a list compiled by Consultancy IQC. They had previously visited these farms and confirmed they were producing eggs in cage-free systems [8]. Two additional farms were purposively recruited to yield additional insights: 1. Company G was selected because it is considered a leader in free-range production with 40 years of experience in this industry, 2. Company I was selected as it was a small, community-led free-range farm, a mode of farming which was otherwise under-represented in the current sample.

Within each company, we recruited 1–2 individuals, resulting in a total of 12 participants. The inclusion criteria for individuals were as follows: 1. they were engaged in a role related to production or sales, and 2. they had worked in the company for at least 1 year.

### 2.3. Data Generation

Data generation took place between 1 August 2022 and 5 September 2023, and involved conducting interviews, collecting relevant documents, and writing reflective field notes. MC interviewed 11 of the 12 participants twice; the remaining participant was interviewed 3 times. Each interview lasted between 0.5 and 2 h and was conducted in Mandarin. Twenty-three of the 25 interviews took place on WeChat; the remaining 2 interviews took place in person at the office of Company C. All interviews were semi-structured, following an interview guide that focused on company background, participant background, products sold, consumers, business buyers, distribution channels, and production methods (see Table S1 in Supplementary Materials). Participants were encouraged to share topics relevant to their work (e.g., a sales representative may prefer to discuss sales rather than egg production).

For each company, MC also gathered publicly available online documents (including text and photos from news articles, online shops, social media content). Where possible, MC conducted in-person fieldwork in Shanghai, China, including visiting 2 participants’ farm, visiting 3 shops where products were sold, purchasing and consuming eggs from 5 companies, and attending 3 industry conferences to meet with 5 participants in person. Throughout, MC took reflective field notes about her experiences and observations. 

### 2.4. Data Analysis 

MC transcribed and reviewed all audio transcripts. All data (interview transcripts, documents, field notes) were organized using NVivo 12.6.0 (QSR International, Vancouver, BC, Canada) for analysis.

We analyzed each case individually (by developing a summary, timeline, and map for each case), while conducting thematic analysis for the entire dataset, enabling both within-case and cross-case comparisons. For individual case analysis, we developed 3 files which provided 1. a case summary (including company background, product sold, major challenges and opportunities related to consumers, business buyers, distribution channels, production methods); 2. a case timeline (a chronology of major events for each case); and 3. a stakeholder map (displaying the relationships between the farms, distribution channels, consumers, and business buyers).

Simultaneously, MC completed template analysis for the entire dataset [27]. This process began with data familiarization, where MC listened to all audio while reading the transcript and making notes. MC then conducted an initial ‘coding’ of a subset of data, whereby ‘coding’ refers to labelling sections of data with a phrase called a ‘code’ (e.g., “consumers like egg samples”). MC highlighted and coded regions in each transcript which were rich in data relevant to the initial research question: “what are the challenges and opportunities to promoting cage-free eggs in China”. Thirdly, MC created an initial ‘template’, which is a table clustering similar codes into categories, for example, “consumers like egg samples” was categorized into “opportunities for engaging consumers”. Codes were clustered descriptively based on the categories they related to: products sold, consumers, business buyers, distribution channels, production methods. Fourth, we refined the template by coding the entire dataset using the initial template while adjusting the template to reflect the data by merging and adding codes. During this iterative process, we generated three themes focused on our finalized research question: “what are strategies used to promote cage-free eggs in China”? To aid analysis, MC kept a reflective document which identified data that were similar, different, contradictory, or surprising, as well as an audit document that recorded major changes to the template and rationale for these changes.

Original Mandarin quotes and the English translations are provided in Table S2 in Supplementary Materials. Findings are summarized into three themes, illustrated using relevant quotes.

## 3. Results

**Figure 1:**
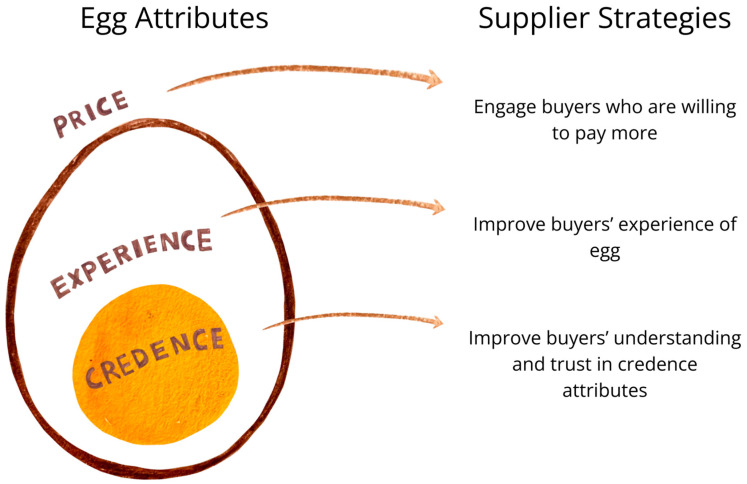
Strategies for suppliers to promote cage-free eggs to buyers (consumers and businesses). These strategies address questions related to egg price, experience, and credence attributes (e.g., cage-free production).

We first share relevant context by introducing the companies, including their production, distribution channels, buyers, and why they sell cage-free eggs. We then describe the strategies used by these companies to sell cage-free eggs, which included addressing the egg’s price (buyer’s purchasing prices), experiential attributes (which can be observed using the buyer’s senses before purchase, e.g., shell color, or observable after purchase, e.g., taste), and credence attributes (which cannot be readily observed by the buyer, e.g., cage-free production system). Specifically, companies promoted cage-free eggs through finding target buyers who were willing to pay a premium for these eggs, aligning their egg products with the experiential expectations of buyers, communicating about credence attributes, and establishing trust with buyers (illustrated in Figure 1).

### 3.1. Context

Participants recognized that ‘cage-free’ meant that hens did not live in cages and that this includes free-range. In practice, participants often referred to indoor systems that did not provide outdoor access as ‘cage-free’ (非笼养) and used the term ‘free-range’ (散养) to refer to systems providing outdoor access. We thus sub-categorized cage-free production into indoor (single or multi-tier) and free-range (see Table 1).

#### 3.1.1. Production

All companies used only cage-free production, except for Companies C, D, and J. C and D each had one cage-free farm representing <1% of their total flock (Company C is a large egg processor who wanted to test the Chinese market’s acceptance of ‘cage-free’ and ‘animal welfare’; Company D is a large egg supplier who received some business buyer demand to produce cage-free eggs). J sourced eggs from multiple caged and cage-free farms. Flock size for companies using indoor systems (15,000 to 110,000 hens) was larger than those providing outdoor access (mainly 6000 to 15,000 hens), except for Company E, which operated free-range farms with a large total flock (80,000 hens). 

#### 3.1.2. Breeds

All companies using indoor systems used commercial layer breeds, which are considered more productive, including Hyline White, Hyline Brown, Dawu and Jinfen-6. In contrast, most companies using free-range systems used local breeds, indigenous to the location of the farm (e.g., the Jianmenguan indigenous chicken 剑门关土鸡). The exceptions were Company E and H which used both commercial and indigenous breeds in outdoor free-range systems. The participants from these companies suggested that it was more productive to use commercial breeds, but also used indigenous breeds to meet consumer demand for pink or green eggs. 

#### 3.1.3. Egg Buyers

Eggs are distributed along the supply chain from suppliers to consumers. We refer to any stakeholder that purchases eggs from these companies (e.g., distributor, trader, retailer, consumers) as an egg buyer, and distinguish between two categories: consumers (individual buyers that consume the egg product) and business clients (buyers along the supply chain who then resell eggs to the final consumer). Companies in this study mainly sold eggs for out-of-home or at-home consumption. Out-of-home consumption included intermediary suppliers and businesses such as hotels, restaurants, canteens in companies, schools, or other businesses sourcing eggs. Several of these buyers had international headquarters that have committed to sourcing only cage-free products. For at-home consumption, eggs were sold offline through retailers including supermarkets, farmer markets, specialty stores, and directly at the farm. Restrictions associated with the COVID-19 pandemic reduced in-person shopping, so companies also developed online distribution channels such as via WeChat stores, group purchases, and Tik Tok. Company A and E sold directly to egg processing companies that made commitments to source cage-free eggs by 2025. 

#### 3.1.4. Motivation for Producing Cage-Free Eggs

Companies chose cage-free systems due to economic and ethical reasons. For economic reasons, companies believed “the product quality is good” (Company G; free-range), that free-range eggs addressed current market demand (“the [free-range] eggs are deeply accepted by consumers”; Company E; free-range), that the cage-free eggs were addressing future demand: “some of our business partners might require the use of cage-free eggs in the future” (Company D; indoor), or to test the market by “bringing the [animal welfare] concept to consumers […] see if it is easily accepted by consumers” (Company C; indoor). Ethically, some participants reported wanting to “produce real free-range eggs, [be] a company that does not lie” (Company E), wanting to “bring real value to society” (Company G; free-range), and to benefit animals: “if we really want chickens to be happy, and lay happy eggs, we must use cage-free system” (Company A; indoor). 

### 3.2. Price: Engaging Buyers Who Are Willing to Pay 

Participants agreed that it was important to engage with buyers who were willing to pay the higher price typically associated with cage-free production. All participants reported a higher cost of production for cage-free compared to caged production; depending on how costs were calculated, cost of production of cage-free eggs was 1.25–3 times more expensive compared cage eggs. Participant G1 described some exceptions including small, free-range farms that produce at a low cost by letting hens forage to reduce feed costs and using cheap and simple infrastructure. Participants also reported high distribution costs, especially when sending small batches of eggs to businesses (e.g., hotels) or individual consumers; these higher costs were reflected in higher prices. 

#### 3.2.1. Consumers’ Willingness to Pay

Retail price varied for different distribution channels, and participants described a large range in retail prices for both caged and cage-free eggs, ranging from 0.4–3 RMB/egg. Cage-free eggs were sometimes sold as regular eggs (at approximately 0.4 RMB/egg) when suppliers had surplus eggs. Various factors were believed to contribute to variation in the price that consumers were willing to pay, including consumer income, intended purpose of the egg, and egg quality (sensory and credence attributes). 

Participants believed consumers were more willing to pay for cage-free eggs when they had more disposable income. Participant C2 (indoor single-tier; eggs marketed as “cage-free”, “animal welfare”; retail price ranging from 2–3 RMB/egg) shared, “Our target audience, […] their family income is relatively high, and they can accept an egg being 3 RMB, rather than 1 RMB”. Similarly, Participant I1 (free-range; eggs marketed as “free-range”; retail price 2.5 RMB/egg) shared, “People who frequently buy [our free-range eggs] have achieved a certain degree of financial freedom. For people from the ordinary working class, it would be difficult to afford this product”. Some companies selected locations where average income is higher, including major cities such as Beijing and Shanghai. Company C (indoor single-tier; eggs marketed as “cage-free”, “animal welfare”; final retail price in the range of 2–3 RMB) sold their eggs at Costco stores in Suzhou and Shanghai (an international retailer which made a 2025 commitment to sourcing cage-free eggs globally). Company C’s eggs were the only cage-free ones in Costco, and the most expensive (2 RMB/egg, double the price of the other eggs sold in Costco). Participant C1 shared: “The sales in Shanghai are better than in Suzhou, mainly because the income level is higher”. 

Consumers who purchased eggs for special purposes (such as for gifts or for children or pregnant women) were reported to be willing to pay more. Participant F1, who produced free-range indigenous chicken eggs and priced these at 2.2 RMB/egg, shared: “In China we still have this sentiment […] what is the most high-end gift for relatives? An indigenous chicken, indigenous chicken eggs, a big basket of it. Especially when someone’s wife is in postpartum confinement period after having a baby, or when there are children”. Suppliers sometimes catered their packaging for gifts; for example, Company H used regular packaging for eggs sold in supermarkets, and “high-end” packaging for gift boxes. Consumers were perceived to be willing to pay more for eggs that they thought were of higher quality; buyer preferences for experiential attributes (e.g., taste) and credence attributes (e.g., free-range production) are discussed in later sections of this paper. 

#### 3.2.2. Business Willingness to Pay

Businesses were reportedly willing to pay more for cage-free eggs if they were pressured to purchase these by their international headquarters, or if they catered to high-income local consumers. As Participant J1 shared, “business clients mainly care about costs”; while individual consumers were willing to pay up to 3 RMB/egg, businesses were generally willing to pay less (at most 1.4 RMB/egg) for cage-free eggs. Our participants reported that these businesses were willing to pay 1.25–2 times more for cage-free eggs compared to caged alternatives, but that price was a main consideration for these business buyers. For example, H1 (free-range eggs sold to consumers through retailers and high-end hotels) shared: “Internationally [some companies] have committed to using cage-free eggs by 2025, but they are still very strict about cost […] last August I signed a contract with [a hotel] in Shanghai, but they only ordered eggs from me twice, 900 eggs each time. I asked him why it was so little; they said the cost was too high. Their current price [for caged eggs] is 0.7 RMB/egg, mine is double at 1.4 RMB/egg. So, though they signed a contract, they only occasionally use our eggs”.

Some businesses that had made cage-free commitments were willing to pay the higher price. H1 shared their experience of supplying eggs to another international five-star hotel: “For example [this hotel] was very reluctant to collaborate with us; they said because their headquarter is in France, they are required to use cage-free eggs, that’s why they chose us, otherwise they would never choose us”. All hotels sourcing cage-free eggs from participants in this study were high-end international hotels that made global cage-free commitments. One participant from Company J reported that all their supply was sold to such hotels. Suppliers such as Company A (indoor) also supplied eggs to Chinese retailers who have made cage-free commitments (e.g., City’super). 

Some companies sold to businesses which catered to high-income local consumers, such as high-end canteens and restaurants. Participant I1 (Company I; free-range) shared: “For canteens, some of our clients are high-end kindergartens, regular kindergartens might not have that purchasing power […] For example, we supply [eggs to] a bilingual school, their students are often children of people from international organizations […] or richer people”. Some locations provided access to high-income consumers; for example, Company A (indoor multi-tier) supplied an egg-themed, high-end brunch restaurant in Shanghai. Participant E1 (free-range) supplied casinos in Macau; these businesses catered to customers who were perceived to be “relatively wealthy” and thus better able to afford these eggs. 

Overall, participants agreed that an important strategy for promoting cage-free eggs was to understand and engage with buyers who were willing to pay for these eggs, for example, either directly to consumers with higher disposable income, or to businesses catering to these higher-income consumers, or to businesses that were committed to buying these eggs. 

### 3.3. Experiential Attributes: Accommodating Buyer Preferences 

Experiential attributes (e.g., egg’s taste, yolk color) can be readily detected by buyers and can affect their willingness to purchase the product. Participants discussed buyers’ preferences for experiential or sensory egg qualities that could be assessed before purchase (e.g., egg size and color) or after purchase (e.g., taste, texture, yolk color). All participants agreed that consumers valued the mouthfeel and taste of eggs. Participant E1 (free-range) shared: “The core [positive] feedback from consumers is mouthfeel. […] The product must be tasty”. Company A (produced indoor eggs marketed as ‘cage-free edible raw’) had salespeople in the supermarkets who promoted their products, and A1 shared that “taste samples [in the supermarkets] had the best effects”.

Participants also shared other preferences, such as shell color, egg size, egg cleanliness, yolk viscosity, and a general preference for darker or non-pale yolk color. For example, F1 (free-range), who raised an indigenous chicken breed that laid a mixture of green, pink, and yellow eggs, shared: “upon opening our egg [packaging], you see mainly green-shelled eggs, with a few yellow and pink ones, right? […] after many clients eat it, they also bought it for their clients or family relatives. […] after you boil the eggs, it has a light fragrance, lots of children really like this. Our indigenous chicken eggs are also smaller than caged eggs”. Participant comments also indicated that consumer preferences were likely heterogenous. For example, C1 shared how consumers preferred pink eggs, but A1 felt that consumers preferred their white eggs, and F1 and I1 believed that their consumers preferred green eggs. 

Participants reported how meeting consumer expectations stimulated purchasing behavior. H1 (free-range) supplied a supermarket in Beijing. He shared: “Although the [egg] brand is not ours, [the product] has our factory address, farm address and phone number on the product certificate, so some consumers call me directly to say: ‘can you deliver eggs to me directly, I will buy a lot […] I want 100 jin (50 kg), for myself, relatives, and friends’ […] They think our eggs’ quality is actually better than eggs that claim to be cage-free, but are actually caged. There is a difference in mouthfeel and other qualities”. Having a detectable difference in the experiential qualities meant some consumers selectively purchased eggs from this supplier. In contrast, C2, who produced eggs using indoor systems, shared that their product did not meet consumers’ experiential expectations for their product. When discussing their low sales, C2 shared: “Many of our consumers feel [our] eggs are not pink shelled, the yolk color is not very bright […] In terms of direct sensory perception, the first impression does not feel good, or perhaps [consumers] may be a bit disappointed. So, it might also affect the [product’s] repurchase, because it’s different from what they traditionally considered good. We are also somewhat hesitant about whether to meet everyone’s psychological expectations, like whether we should change the eggshell color, or adjust the color of the yolk, and so on”. In this quote, the participant reflected and questioned whether they should alter their product to enhance these preferred egg qualities in order to better meet their consumers’ expectations for cage-free eggs.

Some participants described their eggs as being experientially different, while others did not, and of these differences, only some were believed to be associated with cage-free production per se. Participant B1 used an indoor multi-tier system and shared their belief that, all else being equal, “cage free eggs have a distinct advantage in terms of taste and Haught unit (egg thickness, a common measurement of freshness), but the main issue of cage free eggs is the eggs laid outside of the nests are very dirty”. D1, who produced 99% caged and 1% cage-free eggs, conducted a brief experiment comparing eggs from both systems: “The egg white [from cage-free eggs] was slightly thicker […] We did some tests, the Haught unit [for cage free eggs] was higher by around 5% […] But for consumers it is hard to tell [the difference], they might not be able to sense this”.

Participants attributed differences in their eggs to a variety of factors other than the cage-free housing. G1, who produced free-range eggs using indigenous breeds, shared: “Indigenous chicken, as long as they are raised naturally, the egg’s mouthfeel should be good […] It is related to the breed […] their environment […] the nutrition […] their life stage (how old they are)”. He recognized that a combination of these factors contributed to a distinct taste for the eggs and shared positive feedback from children: “Real free-range eggs have a different flavor compared to [eggs] produced in factories. The eating experience is different. For adults, they may have a subjective imagination of ‘I want the taste from childhood’ […] but children are different, they do not have these beliefs and conceptions […] some children never ate chicken and eggs before, but you give them [our] authentic free-range chicken, they will immediately eat it. Because children have very sensitive taste. When they think it tastes good, it means the nutrition is meeting their bodily needs”. Participants recognized that certain egg qualities were unrelated to housing method; for example, shell color and size were impacted by breed. I1 (free-range) shared: “We mainly raise Suqin Grass Chicken, a domestic breed, a regional breed, so compared to caged chickens, there is a difference in the egg, including mouthfeel, eggshell color. Our eggs are pink with some green eggs”. When MC asked if the difference was due to breed or nutrition, I1 clarified “It is related to breed”. 

Participants varied in their approaches to accommodating specific buyer preferences. For example, participant C1 wanted to improve the taste of their eggs: “I am not very pleased with our eggs’ current taste. This is related to the [hen’s] feed. […] We previously did not spend too much energy on taste, but now we want to […] we want the consumers to feel their money is well spent”. In comparison, Participant E1 produced eggs in free-range systems and utilized different hen breeds to meet different demands for egg size and shell color. For mainland consumers, he raised indigenous chicken breeds that produced smaller, brown eggs; for hotel businesses in Hong Kong and Macao, he raised commercial breeds that produced larger, white eggs. E1 shared: “Apart from mouthfeel, [consumers] care about eggshell color. […It can be] hard to negotiate with cage-free [business] buyers, because we raised breeds that lay smaller eggs, which do not meet [business] standards, so we also started raising some breeds [that lay larger eggs]”. 

Participants were hesitant to use feed additives to change yolk color, as they perceived this as unnatural or unethical. For example, I1 (free-range) shared: “Our main point is that we completely raise the free-range chickens on our own […] We purchase grains ourselves, grind them, and then feed the chickens. Throughout the whole process, we would not add anything else. Those large-scale farms [would] increase egg production rates, change the yolk color, adding carophyll red (a synthetic additive which makes yolks redder). We do not do any of these, [we] allow them to lay eggs in a completely natural way”. Similarly, C3 (indoor) was hesitant to add feed additives to change yolk color, as this went against their company’s ethics: “I asked our farm manager, he said we could include [additives to shift yolk color] in the feed [if we wanted to], but we are a company with integrity, we do not want to artificially add these things”.

Overall, participants identified various experiential attributes preferred by buyers, with the priority being taste and mouthfeel for consumers. Participants also shared how cage-free housing was not always associated with experiential differences, and that one strategy to promote cage-free production is to accommodate buyer preferences, for example, by changing hen breed.

### 3.4. Credence Attributes: Improving Buyer Understanding and Trust 

Credence attributes are those which buyers cannot easily detect or assess directly without additional efforts such as visiting the farm (for example, to see how hens are housed). As such, companies needed to communicate these attributes to buyers while establishing trust. We will share how suppliers addressed consumer and business motivations for credence attributes and established trust.

#### 3.4.1. Understanding Buyer Motivation 

Participants reported that consumers were motivated to purchase cage-free eggs based on traditional values (e.g., “free-range 散养”, “indigenous chicken egg 土鸡蛋”), health benefits (e.g., “food safety 食品安全”, “nutrition 营养”), and altruistic motives (e.g., “caring for environment 在意环保” “liking wildlife 喜欢野生动物”). Participants also reported that consumers sometimes associated multiple attributes, for example, that free-range was associated with health benefits (e.g., food safety) and altruism (e.g., liking wildlife). 

All free-range companies reported that free-range and associated concepts such as indigenous chicken eggs (土鸡蛋) were deeply rooted in traditional values. F1, who sold free-range products nationwide, shared: “If you are talking about ecological foods (生态食材) like indigenous chicken eggs […] lots of people from large cities want to buy it, there is a sentiment (情怀) involved in it […] In China, the cities expanded significantly over the past twenty years, and many people in big cities actually have their roots in the countryside, right? […] They still very much miss the countryside’s more carefree or natural lifestyle, and the ingredients and products that come from this way of life”. Thus, consumers in the city were thought to have a nostalgic connection to the countryside, motivating their demand for free-range and indigenous products. 

All participants mentioned the importance of food safety (食品安全) and health (健康). When I1 was asked how he attracted customers for his free-range eggs, he shared: “initially there was the national food safety crisis around 2010 [… and] there were policies stimulating the economy resulting in some people feeling richer, and then caring more about food safety, especially for their children […] The Beijing Organic Farmers Market started then, these people had demand [for free-range eggs]. We just happened to produce these [eggs], it turned out that there was actually no difficulty in selling them at that time”. In this way, I1 suggested that consumers prioritized food safety and associated free-range products with better food safety. 

Company A produced eggs in indoor systems and marketed its eggs as ‘cage-free edible raw’ (非笼养可生食), a term used to imply food safety. When A1 was asked what consumers prioritized when it came to eggs, he said: “food safety and taste”. When asked why consumers bought his cage-free eggs, he replied: “a variety of reasons. Some believe it is tasty, some believe it is healthy. Very few buy because of [animal] ‘welfare production’ (福利养殖). Because people do not have a conception of this yet”. He perceived that consumers were prioritizing the experiential attribute of taste, and credence attributes such as food safety and health.

Some participants perceived that consumers were motivated to purchase their cage-free eggs due to altruistic motives. H1 (free-range) shared how he sold surplus eggs to consumers using environmentally friendly packaging. “These were mainly used for Chinese New Year, festivals, gifts, and sometimes shipped as packaged eggs […] The [consumers] like [the packaging], it is good from an environmental perspective […] most people who order by mail are younger people, they care to a degree about the environment”. Both Company A (indoor cage-free) and Company H (free-range) chose environmentally friendly egg packaging due to positive consumer feedback.

One participant (I1, free-range) shared about consumers who purchased free-range eggs due to their care for wildlife. Company I’s owner was passionate about wildlife and set up cameras showing nearby wildlife, including badgers. I1 shared how some consumers purchased their eggs due to a similar interest in wildlife: “there is one type [of consumers] that, because we keep chickens in the woods, there are some wildlife like badgers, wild-boars, weasels […] the natural environmental is severely damaged, so they do not have much to hunt, so they sometimes steal chicken from our farms. Each year they eat around 200–300 chickens, for our farm of 2000 chickens, almost one chicken lost per day […] [Consumers] like wildlife, so they feel ‘you are keeping chickens, the wildlife eats them, [and] you experience a loss, so we can support you a bit’”. In this case, consumers were reportedly interested in how the free-range production contributed to wildlife conservation and a healthy ecosystem, despite this coming at a cost to the individual hens affected. 

Participants generally shared that consumers had low awareness for ‘cage-free’ (非笼养) and ‘animal welfare’ (动物福利) attributes. Company C only marketed its products with the terms ‘cage-free’ and ‘animal welfare’. C is a large egg processing company, meaning most of its previous work focused on selling and marketing directly to business clients; participants from Company C reported being less familiar with marketing to consumers. It started producing 1% of its eggs from indoor cage-free systems in 2019 to test the consumer market in China and observe if foreign concepts such as ‘cage-free’ and ‘animal welfare’ would be accepted. C2 shared, “Right now Chinese consumers are not familiar with [animal] ‘welfare’ and ‘cage-free’ […] So now there needs to be the process of cultivating [awareness] and education. We are using the term ‘cage-free’ because consumers might understand this, if you really tell them this is a ‘welfare egg’ (福利鸡蛋), many people might not know what it means, why do eggs have welfare. And if you further explain, some people might feel ‘how is the chicken’s welfare relevant to me, why should I pay so much?’” 

#### 3.4.2. Building Buyer Trust 

All participants agreed that consumers had low trust and high skepticism. Information asymmetry between consumers and suppliers, coupled with misleading marketing claims, resulted in consumers not trusting suppliers’ claims. Egg suppliers attempted to increase trust by engaging with consumers, showing production conditions, using third-party assurance, and developing relationships with consumers. 

As an example of low trust, H1 (free-range) shared a prominent incident in China: “The CCTV 3.15 Gala (an annual national TV show in China for promoting consumer rights and addressing market issues), I forgot if it was 2018 or 2019, they suddenly did a session about eggs […] Chinese consumers conceptually equate ‘indigenous chicken eggs’ (土鸡蛋), ‘chai-eggs’ (柴鸡蛋) to ‘free-range eggs’, so they did an undercover investigation of large brands producing ‘indigenous chicken eggs’, but [the companies] were misleading [the consumers] with deliberate falsehoods (混淆视听), they were all producing regular caged eggs (笼养鸡蛋), just calling it ‘chai-egg’, ‘indigenous chicken egg’, then adding some colorants in the feeds to make the yolk darker to deceive the consumers. After that our sales began to plummet…”

Some participants proactively tried to raise awareness of concepts like ‘cage-free’ and ‘animal welfare’. Company A used a variety of marketing techniques, including activities to educate their current potential consumers (urban residents with higher income) and future consumers (children). In October 2023, Company A worked with Consultancy IQC on a series of consumer engagement activities which took place in retail stores across large cities in China. One selected shop was located near the international embassies of Beijing which, as A2 mentioned, meant the visitors had “international perspectives, and an ability to afford [higher priced eggs]”. During the one-day event, salespeople introduced the concept of cage-free to consumers, using creative methods such as a ceremony unleashing helium balloons to symbolize chickens flying out from cages. A2 shared: “the consumers really accepted [the concept], far exceeding our expectations […] That day’s sales [at that supermarket] surpassed the monthly sales volume of other eggs”. A2 further shared: “we can plant the concept and promote cage-free [eggs], starting from the children”. 

Companies showed consumers production conditions through farm visits, videos, and photos. Three companies (H; G; I) operating smaller, free-range farms shared how their consumers valued personal farm visits. H1 (free-range) shared: “Some consumers will personally come to the farm, only after they see it, they will believe this is real”. Similarly, G1 (free-range) spent minimal effort on marketing, sharing that “I feel spending on marketing is not worth it, any time you market, people try to prove if you are real or fake. They always perceive your marketing with skepticism”. Instead, he relies on farm visits. “‘Indigenous chicken egg’, why do I say ‘indigenous chicken’ is a culture? When consumers are in the mountains, in the villager’s homes, when they buy themselves, they believe this is the best”. By encouraging consumers to visit the farms, these free-range companies attempted to improve consumer trust. 

Some companies improved transparency by showing videos and photos of farms to consumers. F1 (free-range) shared: “Additionally, we actually have customers who visit our physical store, and their biggest impression is that we have remote real-time camera monitoring […] Our competitors do not do this, possibly because they do not dare to; because either they are fake, or not entirely genuine, […] So, these are the ways in which we differ from others”. E1 (free-range) also provided live video farm footage to their consumers, through an interactive QR code placed on the product packaging that links to a 24-h live stream of the barn and surrounding free-range area. 

All four indoor companies and four out of the six free-range companies used third-party assurances, such as certifications, awards, or tests displayed on the egg packaging to establish trust with their consumers. For example, E1 had two certifications and two awards. When asked about the benefits of the Certified Humane certification, and whether it was more useful for consumers or business clients, E1 said: “currently, I think it is quite useful for consumers […] The [Certified Humane] certification is international, so Chinese consumers will trust it more”. E1 also believed that the certification worked well in tandem with visual evidence of production methods and a quality product which tastes good. 

Some smaller free-range companies attempted to develop more direct trusting relationships with their consumers. I1 shared: “Some customers come to visit the chicken farm in person, and we have a place where they can stay. Then, they can experience firsthand scenarios like collecting eggs or feeding the chickens. After that, they establish a kind of trust relationship [with us]”. Companies also sought to establish this relationship by prioritizing selling eggs to their loyal consumers, rather than to new clients. “We generally do not like those customers who buy eggs as gifts […] It affects the ordinary consumers […] Those few gifting customers purchase in large quantities, concentrating their orders of eggs in a few days. Then, perhaps our own ordinary customers cannot buy eggs on those days. So, we usually do not take large orders”. 

Participants developed trust with business buyers through communications with decision makers and third-party assurances. Initial communication between suppliers and relevant decision makers in the business took place on an interpersonal level. For example, H1 communicated with the product purchasing manager (采购经理) of a hotel in Shanghai for 1.5 years prior to securing a contract to sell free-range eggs. Multiple participants shared that people within third-party “animal welfare organizations”, such as consultancies (IQC, Lever) or NGOs (Compassion in World Farming), helped them initially connect with business buyers. Participants reported business buyers trusting and even requiring third party certification and tests. For example, Company J was a distributer who supplied eggs from small, free-range farms to local five-star hotels. Prior to receiving certification, J1 shared: “Most of our eggs supplied are cage-free, but right now we have not received the [cage-free] certification, so [even though] we are supplying cage-free, [clients] say ‘without certification, we would not recognize these [as cage-free]”. 

Companies that could not meet stringent requirements were less able to supply to business buyers. Company F reported changing their marketing strategies to not focus on business clients after learning about the stringent requirements. Though consumers and businesses are both concerned about food safety, Company F found his products were sufficient for consumers, but not for businesses. F1 shared how they could not move forward with working with businesses, as “their requirements are too high, like all these safety tests right? […Consumers] are willing to pay the price, and their requirements are not as high, they do not need all these tests […] as long as it is fresh and safe, and it is real ‘indigenous chicken egg’, then they can accept your product”. Companies who supplied successfully to business buyers needed to comply with buyer’s requirements, for example, obtaining relevant certification for cage-free and food safety. 

Participants believed that consumer were motivated to purchase cage-free eggs due to a traditional preference for ‘free-range’ products, health benefits (e.g., food safety), and altruistic motives (e.g., environmentally friendly), and that business buyers were motivated to purchase cage-free products by cage-free commitments and consumer preference for these products. Trust was established through diverse methods for consumers (e.g., showing farm conditions), whereas businesses prioritized third party assurances. 

## 4. Discussion

Our case study examined 10 diverse companies supplying cage-free eggs to consumers and businesses. These results are not intended to be generalizable but may be transferable to situations with similar contexts. Based on our findings, we summarized three main strategies for suppliers to promote cage-free eggs. Firstly, suppliers can engage buyers who are willing to pay more (e.g., consumers who have higher incomes, and those who prioritize the egg’s experiential and credence attributes). Secondly, suppliers can improve the buyer’s experience of the egg (e.g., changing the hen’s breed to match buyer preference for shell color). Lastly, suppliers can improve the buyer’s understanding of relevant credence attributes (e.g., communicating with consumers at retailers) and improve trust (e.g., through providing farm photos or certifications). 

### 4.1. Engaging Buyers Who Are Willing to Pay for a Higher-Priced Product

In the absence of animal welfare legislation, improvements in farm animal welfare are more likely if these provide financial benefits [16]. Despite a higher cost of production and distribution for cage-free eggs (a challenge also identified by other Chinese producers [20,21]), suppliers in this study found buyers who were willing to pay for their eggs. Given that over 95% of eggs in China are consumed as table eggs [2], engaging domestic consumers will likely play an important role in promoting cage-free eggs. Our participants indicated that consumers were willing to pay higher premiums for these eggs than has been reported previously [8,12]. Other work has shown that consumers were willing to pay an increased price for animal welfare-friendly pork products in China [28]. Suppliers in this study targeted consumers with higher incomes, or those motivated to buy higher-quality eggs. Future research can identify characteristics of target consumers of different types of cage-free eggs, and the best distribution channels to reach them. 

Businesses are important drivers in promoting cage-free production [6]. Previous research by Consultancy IQC found that price was rated as an important factor affecting purchasing decisions of business buyers [8], and participants in our study reported a hesitancy by businesses to purchase due to high price. In the current study, suppliers targeted businesses with cage-free commitments and those catering to high-income consumers; successful promotion of cage-free eggs may involve ensuring businesses implement these commitments, for example, through accountability measures [29]. Further research should focus on the perspectives, challenges, and opportunities faced by business buyers when purchasing cage-free eggs and the support businesses may need, and highlight cases where progress has been made, such as those mentioned by participants in the current study (including international businesses like IKEA and Marriot, and Chinese businesses like City’super). 

### 4.2. Accommodating Buyer Preferences for Sensory Qualities 

Sensory properties are major determinants of egg purchases by consumers [30]. Participants in this study reported a nuanced relationship between cage-free production and sensory characteristics. Participants acknowledged that egg qualities were influenced by a variety of factors beyond housing systems [31], including factors related to the hens (genetics, age; [32,33]), management (e.g., nutrition; [34,35]), environment (e.g., season; [36]), egg collection and storage [37]. While some studies reported no difference in egg quality between housing systems [38], other studies reported that cage-free housing is associated with changes in egg quality [39], and some changes (for example, in yolk color) may be attributed to changes in the hen’s diet in outdoor systems [35,40]. Some other changes, for example, in eggshell strength [41], may be difficult for buyers to detect, and slight changes in Haught unit (egg white viscosity; [42]) or egg weight [43] may be affected by factors other than production method, such as egg storage and hen breed. Thus, caged hens can produce eggs with sensory experiences similar to those from cage-free hens.

Participants highlighted the importance of taste and mouthfeel to consumers, and these attributes can be modified by changes in hen nutrition [34,44]. Our results contrast with those of an earlier study that reported that livestock leaders in China rated taste as the least important consideration for products [16], but our work agrees with that from previous studies showing that taste is important to consumers [45]. Suppliers utilized taste as a consumer engagement strategy, for example, using free samples and taste tests at retail stores to engage consumers. 

Our participants reported variable preferences for egg characteristics, in line with previous research. For example, Chen et al. [46] found that consumer preference for shell color and yolk color varied depending on region, gender and age. For example, men and consumers in the Northeast preferred darker shell color, and older consumers preferred darker yolks. Given the diversity of consumers within China, further research is required to better understand these diverse preferences and better meet the needs of these consumers. 

### 4.3. Improving Buyer Trust in Relevant Credence Attributes 

Previous research found that while some consumers are likely most concerned about price, others were more concerned with credence attributes such as food safety or production methods [22], reflecting a general rise in consumer concern [47]. Knowledge and belief about egg products can affect purchasing behavior [30]. Internationally, consumers are known to prefer cage-free eggs over caged eggs due to the perception of higher hen welfare, and associations with health, food safety, and sensory attributes [48,49]. In China, lack of consumer familiarity with the term ‘cage-free’ was identified as a challenge by participants of our study and in other studies [20,21]. Consultancy IQC found that as many as 80% consumers surveyed were unfamiliar with the term ‘cage-free eggs’ 非笼养鸡蛋 [8], possibly because this term was a literal translation from English. We do not believe that this lack of familiarity should be taken as evidence of a lack of concern, as other work has shown concern in China about the treatment of farm animals [50] and animal welfare [28,51]. 

Understanding the underlying motivations of consumers who purchase cage-free eggs may help suppliers focus their marketing efforts. Though industry stakeholders appeared to link animal welfare with cage-free production [8,10], participants in this study perceived that consumers were generally unaware of, or uninterested in, animal welfare and its link to cage-free production. They viewed consumers as motivated by concerns regarding their own health, food safety, and benefits to the ecosystem; these reasons are similar to those motivating consumers buying organic products [52,53]. Despite the late start to the market in China, and information asymmetry between sellers and consumers [54], China is now one of the largest organic markets in the world [55]. The target audience for cage-free eggs may include those interested in organic products; for example, one study found that younger and highly educated consumers reported a higher intent to purchase organic tea [56]. Another study found that consumers with more education and higher food expenditures, women, children, and the elderly were more concerned with food safety [57], and another study found that buyers who were younger, higher educated, and had higher incomes were more interested in high-welfare pork [28]. Thus, even though cage-free and animal welfare are novel concepts in China, marketing research can build upon existing knowledge to communicate these concepts in a way which aligns with known consumer concerns and values.

Chinese consumers often have low trust in the food system [58], especially after multiple food safety scandals in the 2000s [59], and for eggs, after the 2019 exposé of companies selling ‘fake’ free-range eggs [60]. Food safety was reported as important by all participants in this study, and an earlier study also found that Chinese consumers ranked this as the most important quality, even more so than nutrition, appearance, price, and brand [46]. In this context, suppliers need to prioritize food safety. Beyond food safety, consumers show a preference for free-range products [11,12,60], but this requires trust. Suppliers can work to establish trust in multiple ways, including via official certification schemes. Multiple suppliers in this study obtained Certified Humane and Cage Free labels. The results of previous studies on Chinese consumers’ trust in labelling were mixed, with some studies suggesting consumer trust in official certifications and others suggesting more skepticism [60]. Some consumers resorted to using their senses to establish trust, for example, visiting farms and tasting the eggs [60]. Alternative food networks such as farmer markets and community-supported agriculture have developed in China as a method to reestablish trust [61], but these strategies were used in this study mainly by companies using free-range rather than indoor systems; as consumers may be less familiar with indoor systems, suppliers may need to navigate the best ways to ensure trust, potentially through leveraging consumer trust in channels like high-end supermarkets. 

### 4.4. Strengths and Limitations 

A strength of this study was that the study participants were diverse, enabling us to compare suppliers with different flock sizes, housing systems, distribution channels, and breeds. We focused on farms with smaller flocks, too, in contrast with previous studies focusing mainly on larger flocks (e.g., Yang’s [20] study required a minimum of 10,000 birds, De Luna et al.’s [21] study required flocks of 50,000 or more birds). Our study also examined participants using both commercial and indigenous breeds, as there is a preference for eggs from indigenous chickens in China [13]. Multiple data collection methods allowed triangulation [62], for example, by purchasing and consuming the egg from one company, MC was able to ask relevant questions regarding taste and yolk color in a follow-up interview; when participants’ reports differed from MC’s observations in shops or farms, these points prompted further discussion. Additionally, repeated interviews allowed MC to build rapport and ask follow-up questions. 

One limitation of our study is that we only spoke to one or two participants from each company, with participants usually specializing in production or sales (a few smaller companies had people who oversaw both). Additional interviews with employees with different roles within the same company would have been beneficial. 

## 5. Conclusions

For layer hens, cage-free housing provides a potential for improved welfare outcomes. As China is mainly self-sufficient in egg production and consumption, demand for cage-free eggs from both business and consumer buyers in China acts as an important driver for suppliers to use cage-free housing. We found suppliers using cage-free housing in China were motivated to use these housing systems for both economic and ethical reasons. To promote cage-free products to consumer and business buyers, participants targeted buyers who were willing to pay more, accommodated buyers’ preferences for the sensory egg experience, and established trust in credence attributes such as cage-free, animal welfare, and free-range. Cage-free housing refers simply to the absence of cages, and there are a wide variety of cage-free systems, each with unique welfare benefits and concerns. Additionally, the housing system is only one factor among many which affect the welfare of animals; hen genetics, staff competence, climate and more can result in different welfare conditions within the same housing. As cage-free systems cannot guarantee high animal welfare, promotion of cage-free housing and cage-free eggs needs to happen together with efforts to ensure that hens benefit from these systems.

## Figures and Tables

**Table 1:** Company context. Overview of production and distribution of cage-free eggs. For each participating company, we show the percentage of their egg products produced in cage-free systems (% CF), total cage-free (CF) flock size, housing method, breed classification (classified by participants as ‘foreign commercial’, ‘Chinese commercial” or ‘local breed’), and distribution channels. The table is separated by system (indoor or outdoor), and within each category is sorted by flock size.

Company	% CF	Total CF Flock Size	Housing	Breed	Distribution Channel
**Indoor**					
A	100%	110,000	Multi-tier barn	Foreign commercial	Consumers (retailers, online); Businesses (restaurants, hotels, etc.)
B	100%	40,000	Multi-tier barn	Chinese commercial	Consumers (offline)
C	<1%	25,000	Single tier barn	Foreign commercial	Consumers (retailers; online)
D	<1%	15,000	Multi-tier barn	Chinese commercial	Consumers (retailers; online)
**Outdoor**					
E	100%	80,000	Free-range	Foreign commercial; Local breed	Consumers (retailers; online); Businesses (hotels; casinos)
F	100%	15,000	Free-range	Local breed	Consumers (own store; online)
G	100%	8000	Free-range	Local breed	Consumers (online)
H	100%	8000	Free-range	Foreign commercial; Local breed	Consumers (retailers; online); Businesses (hotels)
I	100%	6000	Free-range	Local breed	Consumers (farmers’ market; online); Businesses (schools)
J	60%	6000	Free-range	Local breed	Businesses (five-star hotels)

## Data Availability

The data presented in this study are available on request from the corresponding author. Sharing the anonymized data may still pose a risk that companies and participants are identified through contextual descriptions, and participants did not consent to have their full transcript data made available.

## Supplementary Materials

The following are available online at https://www.mdpi.com/article/10.3390/ani14111625/s1, Table S1: Interview guide, Table S2: Mandarin quotes and English translations.
